# Atkinson Resolutions.—St. Louis Dental Society

**Published:** 1891-04

**Authors:** Wm. Conrad, Wm. H. Eames, J. B. Newby

**Affiliations:** Committee.; Committee.; Committee.


### ATKINSON RESOLUTIONS—ST. LOUIS DENTAL SOCIETY.

We, your committee, appointed to present resolutions of respect to the memory of the late Dr. Wm. H. Atkinson, New York City, beg leave to offer the following:

WHEREAS, The members of the St. Louis Dental Society have learned with deep regret of the sad death of one who has been so closely identified with every advance made by the dental profession during the past twenty-five years; whose honorable career as a professional man has won for him a world wide reputation, and whose personal qualities secured for him the love of every reading dentist in the world.

*Resolved*, That the St. Louis Dental Society recognizes the obligation dentists of America owe to the late Dr. Wm. H. Atkinson for the zeal and energy with which he has advocated the many changes which have been for the elevation of his chosen profession.

*Resolved*, That as a mark of appreciation of the worth of the late Dr. Wm. H. Atkinson, as a man and dentist, these resolutions be spread upon the records of this Society, a copy be sent to the family, and to the dental journals for publication.

WM. CONRAD,
WM. H. EAMES, } *Committee.*
J. B. NEWBY,

